# Pertussis Vaccine Candidate Based on Outer Membrane Vesicles Derived From Biofilm Culture

**DOI:** 10.3389/fimmu.2021.730434

**Published:** 2021-09-15

**Authors:** Francisco Carriquiriborde, Pablo Martin Aispuro, Nicolás Ambrosis, Eugenia Zurita, Daniela Bottero, María Emilia Gaillard, Celina Castuma, Erika Rudi, Aníbal Lodeiro, Daniela F. Hozbor

**Affiliations:** ^1^Laboratorio VacSal, Instituto de Biotecnología y Biología Molecular (IBBM), Facultad de Ciencias Exactas, Universidad Nacional de La Plata, CCT-CONICET La Plata, La Plata, Argentina; ^2^Instituto de Biotecnología y Biología Molecular (IBBM), Facultad de Ciencias Exactas, Universidad Nacional de La Plata, CCT-CONICET La Plata, La Plata, Argentina

**Keywords:** *Bordetella pertussis*, outer membrane vesicles, biofilm, planktonic, protection, vaccine

## Abstract

Outer membrane vesicles (OMV) derived from *Bordetella pertussis—*the etiologic agent of the resurgent disease called pertussis—are safe and effective in preventing bacterial colonization in the lungs of immunized mice. Vaccine formulations containing those OMV are capable of inducing a mixed Th1/Th2/Th17 profile, but even more interestingly, they may induce a tissue-resident memory immune response. This immune response is recommended for the new generation of pertussis-vaccines that must be developed to overcome the weaknesses of current commercial acellular vaccines (second-generation of pertussis vaccine). The third-generation of pertussis vaccine should also deal with infections caused by bacteria that currently circulate in the population and are phenotypically and genotypically different [in particular those deficient in the expression of pertactin antigen, PRN(-)] from those that circulated in the past. Here we evaluated the protective capacity of OMV derived from bacteria grown in biofilm, since it was observed that, by difference with older culture collection vaccine strains, circulating clinical *B. pertussis* isolates possess higher capacity for this lifestyle. Therefore, we performed studies with a clinical isolate with good biofilm-forming capacity. Biofilm lifestyle was confirmed by both scanning electron microscopy and proteomics. While scanning electron microscopy revealed typical biofilm structures in these cultures, BipA, fimbria, and other adhesins described as typical of the biofilm lifestyle were overexpressed in the biofilm culture in comparison with planktonic culture. OMV derived from biofilm (OMVbiof) or planktonic lifestyle (OMVplank) were used to formulate vaccines to compare their immunogenicity and protective capacities against infection with PRN(+) or PRN(-) *B. pertussis* clinical isolates. Using the mouse protection model, we detected that OMVbiof-vaccine was more immunogenic than OMVplank-vaccine in terms of both specific antibody titers and quality, since OMVbiof-vaccine induced antibodies with higher avidity. Moreover, when OMV were administered at suboptimal quantity for protection, OMVbiof-vaccine exhibited a significantly adequate and higher protective capacity against PRN(+) or PRN(-) than OMVplank-vaccine. Our findings indicate that the vaccine based on *B. pertussis* biofilm-derived OMV induces high protection also against pertactin-deficient strains, with a robust immune response.

## Introduction

Pertussis, a highly contagious respiratory disease mainly caused by the Gram-negative bacterium *Bordetella pertussis*, has resurged in many countries even in those with high vaccination coverage (1–5). In order to confront this worrying epidemiological situation in the short term, vaccination schedules have been modified in many countries by implementing additional boosters (6–8). Currently, whole cell vaccines (wP) based on standardized cultures of *B. pertussis* strains and acellular vaccines (aP) composed of two (pertussis toxin and filamentous hemagglutinin), three (pertussis toxin, filamentous hemagglutinin and pertactin) or five (pertussis toxin, filamentous hemagglutinin, pertactin and fimbriae-2 and -3) immunogens are available. However, these vaccines manifest some weaknesses, such as the reactogenicity associated to wP or the faster waning immunity induced by aP (9, 10). This situation requires a third generation of vaccines with the capacity to overcome such weaknesses in the medium-long term (11). Thus, this new generation of vaccines must be i) safer than wP, ii) able to induce an immune response profile that is mostly Th1 and Th17 (12) with proliferation of the memory cell population resident in tissues (13, 14), iii) made up of multiple epitopes to minimize the selection pressure that it may exert on the circulating bacterial population, iv) able to protect against the circulating bacterial population, and v) biotechnologically easy to produce, ensuring accessibility to the entire population (5, 15).

The World Health Organization recommends, in Annex 6 of vaccines production against pertussis, that the strain used (hereafter referred to as vaccine strain) must be characterized and have a known history (https://www.who.int/biologicals/publications/trs/areas/vaccines/whole_cell_pertussis/Annex%206%20whole%20cell%20pertussis.pdf?ua=1visited on June 15 of 2021). Under this context, we designed a novel acellular vaccine candidate based on OMV derived from *B. pertussis*. This candidate was obtained from the strain *B. pertussis* Tohama phase I from the Collection of the Pasteur Institute in Paris (France), whose genome was sequenced and whose genotypic and phenotypic characteristics were widely studied by different laboratories (16–18). This strain was isolated in 1954 in Japan and since then, it has been used to produce wP and more recently to obtain immunogens that constitute aP. We already showed that OMV derived from *B. pertussis* Tohama phase I are safe and effective in preventing bacterial colonization in the lungs of immunized animals (19, 20). Furthermore, we have shown that vaccine formulations containing OMV derived from *B. pertussis* are capable of inducing a mixed Th1, Th2 and Th17 profile, but even more interestingly, they may induce a tissue-resident memory immune response (13). More recently, we described that these OMV induce inflammasome by the canonical and non- canonical ways (21). In addition, the OMV-based vaccine may protect against modern circulating isolates that possess the *ptx*P3, *prn*2 and *ptx*A1 genotypes (10, 22). The current challenge, however, is generating protection against strains that do not express the pertactin (PRN) vaccine antigen. PRN-deficient *B. pertussis* strains have recently been detected as prevalent bacteria in countries using aP vaccines (e.g. United States: 85%, Australia:> 80%, Sweden: 69%, etc.) (23–25). On the contrary, the prevalence of PRN-deficient *B. pertussis* strains in countries that switched to aP vaccines that exclude pertactin in their composition or use wP vaccine to cover the primary vaccination series, is low. As examples, Japan reduced the prevalence of PRN-deficient *B. pertussis* strains from 41% to 8% (26) and Argentina has very rare detection of PRN-deficient *B. pertussis* strains (27). All these observations provide a strong correlation between the use of aP vaccines containing PRN and a higher prevalence of PRN-deficient strains, suggesting a lower protective capacity of such aP vaccines against PRN-deficient *B. pertussis* in comparison with that conferred by wP vaccine. Furthermore, experiments using the murine model of protection have shown that PRN-deficient strains are more resistant to the immunity induced by aP vaccines than those expressing PRN (28, 29).

To further improve the protective capacity of OMV against the newest PRN(-) genotypes, one possible strategy is to obtain OMV from currently circulating clinical isolates. Since bacteria continue to evolve with a bottleneck caused by vaccines selective pressure, this strategy would force us not only to continuously select a representative clinical isolate but also to establish the frequency of this change. A rational alternative strategy is to identify a common and specific characteristic among the clinical isolates not present in the strains used for vaccine production. After decades of laboratory adaptation, vaccine strains evolved for planktonic growth in culture media under controlled conditions. By contrast, biofilm growth is detected in a much larger extent in clinical isolates—which present the characteristic of having recent contact with the host—than in laboratory-adapted strains (30). The formation of biofilms has been shown to increase the virulence and persistence of *B. pertussis* in the human nasopharynx (31). Furthermore, it has been shown that membrane proteins derived from the *B. pertussis* biofilm have immunogenic characteristics with protective capacity against virulent infection by *B. pertussis* in the murine protection model (32–34). At this point it is important to underscore that all currently available pertussis vaccines are formulated with antigens derived from planktonic bacterial cells. Under this context, we turned our efforts towards the investigation of OMV obtained from *B. pertussis* biofilms as an improved vaccine candidate. In this study we have evaluated whether a vaccine formulated with those OMV is capable of overcoming the deficiencies of commercial vaccines in both controlling infections caused by the PRN(-) isolate/strain and inducing the recommended immunity for protection.

## Materials and Methods

### Animals

Animal experiments were performed using female BALB/c mice 3 to 4 weeks of age, obtained from Faculty of Veterinary Sciences of the National University of La Plata (UNLP, Argentina). The studies have been approved by the Ethical Committee for Animal Experiments of the Faculty of Science of UNLP (Argentina, approval number 004-06-15 and 003-06-15).

### Bacterial Strains and Growth Conditions

*B. pertussis* Tohama phase I strain, the Argentinian clinical isolate BpAR106 collected in 2001 from an infant patient residing in Buenos Aires (35), and PRN-deficient clinical isolates from both 2012 Washington (US) outbreak (36) and Argentinian collection of our Reference Laboratory (27) were used throughout this study. PRN deficiency in this isolate was caused by IS481 insertion in its *prn* gene, and its MLVA-MLST type is the most prevalent among the isolates from that outbreak (*ptx*P3, *ptx*A1, *prn*2).

Bacteria were grown on Bordet-Gengou agar (BGA, Difco) supplemented with 10% defibrinated sheep blood at 36.5°C for 72 h and plated again in the same medium for 24 h before each infection.

To carry out the planktonic culture of *B. pertussis*, the biomass obtained after replating for 24 h on BGA was used as inoculum for the Stainer-Scholte (37, 38) liquid medium supplemented with 1% casaminoacids (SSC). This inoculated medium was incubated at 36.5°C with shaking at 160 rpm until the optical density at 650 nm reached 1.2. For biofilm cultures, a planktonic culture was used as starting biomass and petri dishes as abiotic support. These petri dishes containing SSC medium were incubated at 36.5°C under static conditions for 96 h. To evaluate the biofilm formation, a modified version of the crystal violet assay described by O`Toole (39) was performed. At the incubation endpoint, the plates were washed out using PBS (pH 7.2) to remove the planktonic bacteria. Attached bacteria were then stained with 1 mL of 0.1% crystal violet solution (Biopack). The stain was dissolved by adding 1000 µL of 33% acetic acid solution. One-hundred µL of the stain solution was transferred to flat bottom microplates and quantified by measuring at 595 nm.

The biomasses from both planktonic and biofilm cultures were used to obtain outer membrane vesicles (OMV) as described below.

### Isolation and Characterization of Outer Membrane Vesicles (OMV)

OMV were produced and characterized as previously described (40). Briefly, samples from the decelerating growth phase in liquid medium or 96 h-biofilm cultures were centrifuged and the bacterial pellets were resuspended in 20 mM Tris–HCl, 2 mM EDTA, pH 8.5. The suspension was sonicated (ultrasonic bath) in cool water for 20 min. After two centrifugations at 10,000 ×*g* for 20 min at 4°C, the supernatant was pelleted at 100,000 ×*g* for 2 h at 4°C. This pellet was resuspended in 20 mM Tris–HCl, pH 7.2. The samples obtained were negatively stained for electron microscope examination and size estimation. Protein content was estimated by the Bradford method using bovine serum albumin as standard (41). The presence of the main immunogenic proteins in the OMV was corroborated by immunoblot assays using specific antibodies as previously described (20, 42).

### Scanning Electron Microscopy

The biomass obtained in the biofilm culture was fixed on the abiotic support by adding 2.5% glutaraldehyde. Increasing alcohol solutions ranging from 20 to 100% were used for dehydration. For observation, the sample was dried by the critical point technique and coated in gold. This procedure was performed by the microscopy service of the Metallurgical Physical Research Laboratory Ing. Gregorio Cusminsky (LIMF) from the Faculty of Engineering of UNLP, the equipment used for the observation was the FEI Quanta 200.

### Transmission Electron Microscopy - Negative Staining

Transmission electron microscopy was carried out with a suspension of OMV in 0.1 mM ammonium acetate, pH 7.0. A drop of this suspension was placed on a grid covered with a reinforced carbon film. After 30 seconds, the excess liquid was gently removed with a filter paper and the grid was stained with a 2% phosphotungstic acid, pH 5.2. Observations were done with a Jeol JEM 1200EX Microscope.

### Sodium Dodecylsulfate-Polyacrylamide Gel Electrophoresis (SDS-PAGE)

Samples of OMV obtained from *B. pertussis* cells were treated with Laemmli sample buffer and run on 12.5% SDS gels as previously described (42). Electrophoresis was performed at room temperature and constant voltage. Polypeptides and lipo-oligosaccharides were visualized by Coommassie Blue- and the BioRad silver-staining techniques, respectively.

### Comparative Proteomic Study Between Samples Obtained From Planktonic and Biofilm Cultures

For this study, the methodology described by Nilsson et al. (43) was performed with the BpAR106 isolate. Briefly, 3 biological replicates of a bacterial lysate from a biofilm culture and another 3 from a planktonic culture were used. To obtain the samples, a bacterial suspension of OD650nm = 20 was started, which was then subjected to rupture using a Rybolizer Precellys equipment. After eliminating the cellular debris, a precipitation was carried out with 100% trichloroacetic acid at 4°C ON. The sediments obtained from this precipitation were resuspended in 50 mM ammonium bicarbonate and treated with trypsin (Promega V5111). The trypsinized samples were desalted with Zip-Tip C18 columns (Millipore) and injected on a Nanoflow Ultrahigh-performance Liquid Chromatography (Thermo Scientific, model EASYnLC 1000) with an Easy-spray column (P/N ES801, C18, 2 μm, 100 Å, 50 μm × 15 cm, serial number: 10433446, temperature 50°C, Thermo Scientific) coupled to a quad Orbitrap mass spectrometer (Thermo Scientific, model Q-Exactive). The threshold for ion precursor selection was 10,000. The number of precursors chosen in each scan cycle was 15, and the accepted mass window for this selection was 1.6 m/z. The collision energy was normalized at 27. These analyzes were carried out at the Center for Chemical and Biological Studies by Mass Spectrometry (CEQUIBIEM), of the University of Buenos Aires (UBA).

For the analysis of the mass spectra and the identification of the peptides, the Max Quant software (version 1.5.3.30) was used. The search was carried out using the information on the genomic sequences of *B. pertussis* present in the Uni-Prot database. The identification of the peptides was carried out with a falsehood range (FDR) of 1% and the peptides thus selected were linked to a protein with a FDR also of 1%. The search parameters allowed up to 2 erroneous cuts with trypisin/P in the sequence and oxidation of methionine. In the identification of the proteins, a minimum of 2 peptides was used; the quantification of unlabeled proteins was performed as found with the MaxQuant software (version 1.5.3.30). All biological replicas of both culture conditions were included in the experimental design of the quantification of the level of label free proteins (LFQ) and normalization was carried out with 0.01 and 0.05 FDR filters for peptides and proteins respectively. For the statistical analysis, the Perseus program (version 1.5.6.0, from the Max Planck Institute of Germany) was used. The significance of the changes between biofilm and planktonic growth was determined with the Student’s *t*-test with a *p* value <0.05.

### Formulation of OMV-Based Vaccine

The characterized OMV from planktonic or biofilm growth conditions that range in size from approximately 50 to 200 nm in diameter were used to formulate the vaccine in suboptimal quantity for protection (0.75 µg total protein per dose) with tetanus (5 to 7 Lf/dose with a power greater than or equal to 2 UIA/ml serum) and diphtheria (1 to 3 Lf/dose with an output of 0.1 UIA/ml serum) toxoids as previously described. The safety of this vaccine was confirmed by human whole-blood IL-6–release assays. The results were presented in **Figure S1** of **Supplementary Material**.

### Immunization of Mice

Groups of 4-5 female BALB/c mice were immunized with OMV-based vaccine formulated as previously described (0.75 µg total protein per dose) using a two-dose schedule. For protection assays mice were challenged with sublethal doses (10^7^-10^8^ CFU/40 μl) of *B. pertussis* BpAR106 PRN(+), USA PRN(-) *B. pertussis* clinical isolate, or Argentinian PRN(-) *B. pertussis* clinical isolate. Mice were sacrificed 1 week after challenge and bacterial counts from the lungs of treated animals were performed as we previously described (20).

### Enzyme-Linked Immunosorbent Assay (ELISA)

As we previously described (44), plates (Nunc A/S, Roskilde, Denmark) were coated with sonicated *B. pertussis* BpAR106 (whole-cell lysates) at 3 µg/ml in 0.5 M carbonate buffer, pH 9.5, by means of an overnight incubation at 4°C. The rinsed plates were then blocked with 3% milk in PBS (2 h at 37°C) and incubated with serially diluted samples of mouse serum (1 h at 37°C). In the experiments described above, blood samples were collected and the sera were obtained after leaving the blood samples to clot for 1 h at 37°C followed by centrifuging for 10 min at 7,000 × *g*. IgG from individual serum or pooled sera bound to the plates were detected after a 2-h incubation with goat anti–mouse-IgG–linked horseradish peroxidase (1:8,000 Invitrogen, USA). For measuring IgG isotypes, detection of bound antibodies was performed using HRP labeled subclass-specific anti-mouse IgG1 (1:8,000) or IgG2a (1:1,000) (Sigma, Aldrich). As substrate, 1.0 mg/ml o-phenylendiamine (OPD, Bio Basic Canada Inc) in 0.1 M citrate-phosphate buffer, pH 5.0 containing 0.1% hydrogen peroxide was used. Optical densities (ODs) were measured with Titertek Multiskan Model 340 microplate reader (ICN, USA) at 492 nm, and the OD was plotted as a function of the log of the inverse of serum dilution factor. A successful assay produced, for each antibody sample, a sigmoidal curve in this type of plot. The titer of each antibody sample was determined from this curve by identifying the concentration by GraphPad Prism^®^ software (expressed as the inverse of the dilution factor of the antibody) that provokes a half-way between the basal and maximal responses.

From the experimental protocol performed in triplicate, one representative experiment is presented in the Results.

### Avidity Assay

Avidity was measured by an ELISA elution assay as the overall strength of binding between antibody and antigen, using plates incubated for 15 min with increasing concentration of ammonium thiocyanate (NH_4_SCN) from 0 to 0.375 M. Antibody avidity was defined as the amount (percentage) of antibody eluted for each increment of NH_4_SCN concentration.

### Statistical Analysis

For the analysis of CFU counts in animal lungs, we evaluated the normality of the data by using Shapiro-Wilk test (http://scistatcalc.blogspot.com.ar/2013/10/shapiro-wilk-test-calculator.html) before applying the statistical methods described below. After verifying that our CFU data follows a normal distribution, we analyzed them by one-way analysis of variance (ANOVA) followed by Bonferroni´s multiple comparison test. Differences were considered to be significant when *p*<0.05. All statistical analyses were performed using GraphPadPrism^®^ version 6.00 for Windows, GraphPad^®^ Software.

## Results

### *B. pertussis* BpAR106 Growth in Biofilm Culture Condition

**Figure 1:**
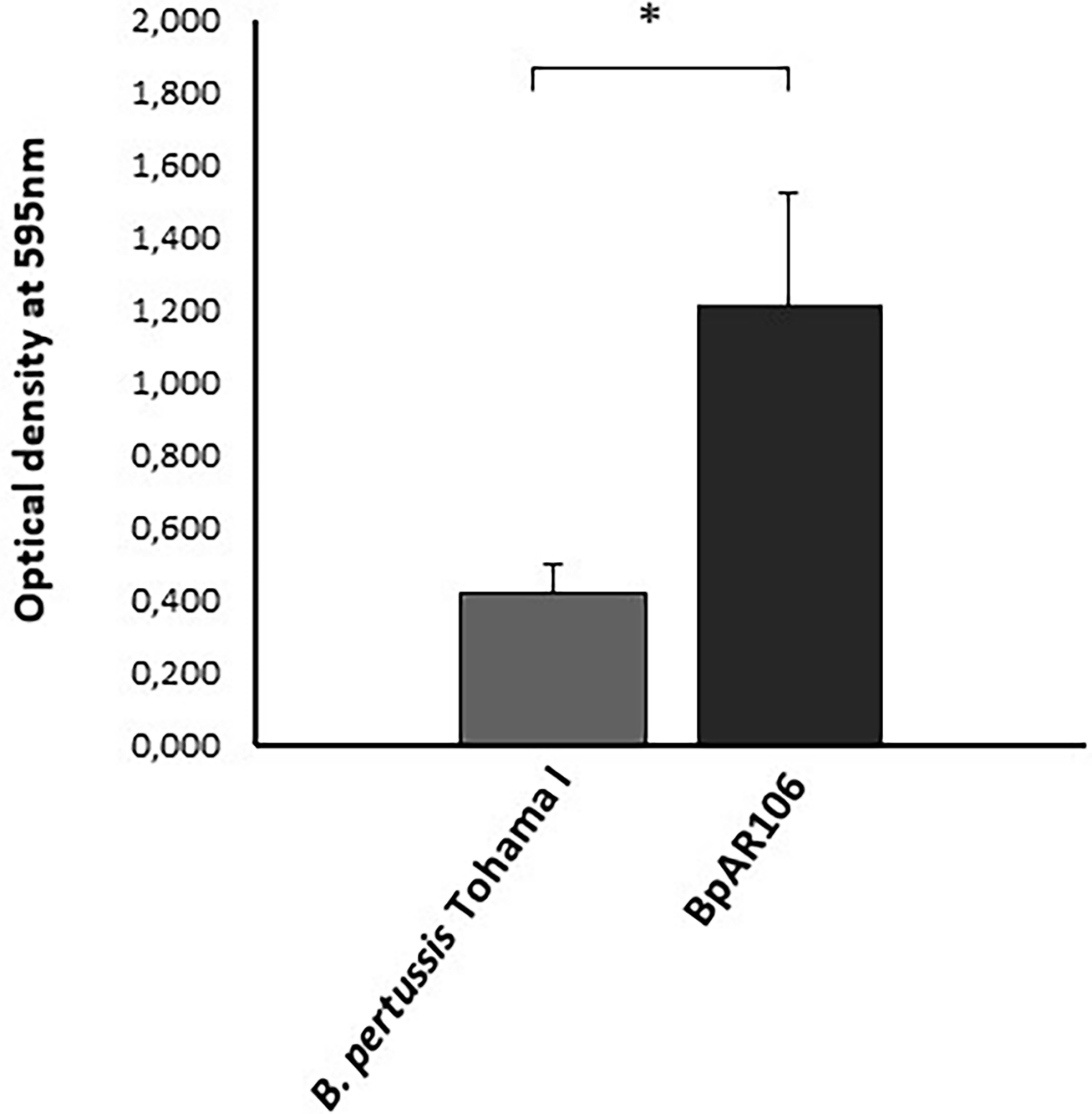
Biofilm formation by *B. pertussis* Tohama I strain (gray column) and the clinical isolate BpAR106 (black column) on an abiotic surface. Biofilm biomass was quantified at 96 h of growth by staining with crystal violet and absorbance was measurement at OD 595 nm. Each value represents the mean from three independent experiments and the bars indicate standard deviation. Statistically significant differences in Student’s t-test between the clinical isolate and *B*. *pertussis* Tohama I biomass absorbance are indicated by * (p < 0.05).

Based on a relatively recent report on the higher capacity of Argentinian *B. pertussis* clinical isolates to form biofilms in comparison with reference strains adapted to laboratory growth condition (30) we decided to perform this study with the BpAR106 clinical isolate, which was sequenced and characterized by our group, possesses the *ptx*P3, *ptx*A1, *prn*2 genotype, and expresses pertactin (45). We evaluated the ability of BpAR106 to form biofilm in petri dishes in comparison with the reference strain *B. pertussis* Tohama phase I. Negative controls consisted of uninoculated petri dishes containing sterile medium only. In agreement with a previous report (30) BpAR106 exhibited more than twofold mature biofilm biomass after 96 h of culture compared with the reference strain *B. pertussis* Tohama I (**Figure 1**).

**Figure 2:**
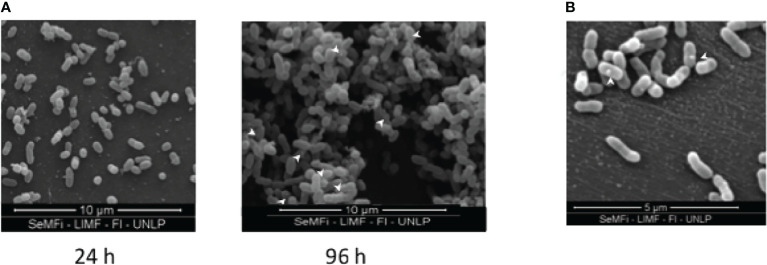
**(A)** Scanning electron microscopy of *B*. *pertussis* BpAR106 biofilm culture on abiotic surface at 24h and 96h of growth. For observation through the FEI Quanta 200 electron microscope (24,000 x magnification), the samples were dried by the critical point technique and coated in gold. **(B)** To visualize the OMV, an electronic zoom of panel **(A)** is shown. White arrows were used to mark the presence of OMV.

The formation of a biofilm (pellicle) at the air-liquid interface for BpAR106 was analyzed by scanning electron microscope observations. While at 24 h of incubation bacteria looked dispersed on the surface (**Figure 2A**), at 96 h of culture the bacteria adhered to each other, forming a complex internal architecture characteristic of biofilm growth. In addition, bacteria were stacked forming three-dimensional towers separated by bacteria-free furrows. Through these electron microscopic observations, the presence of OMV in the biofilm condition was detected (**Figure 2B**).

**Figure 3:**
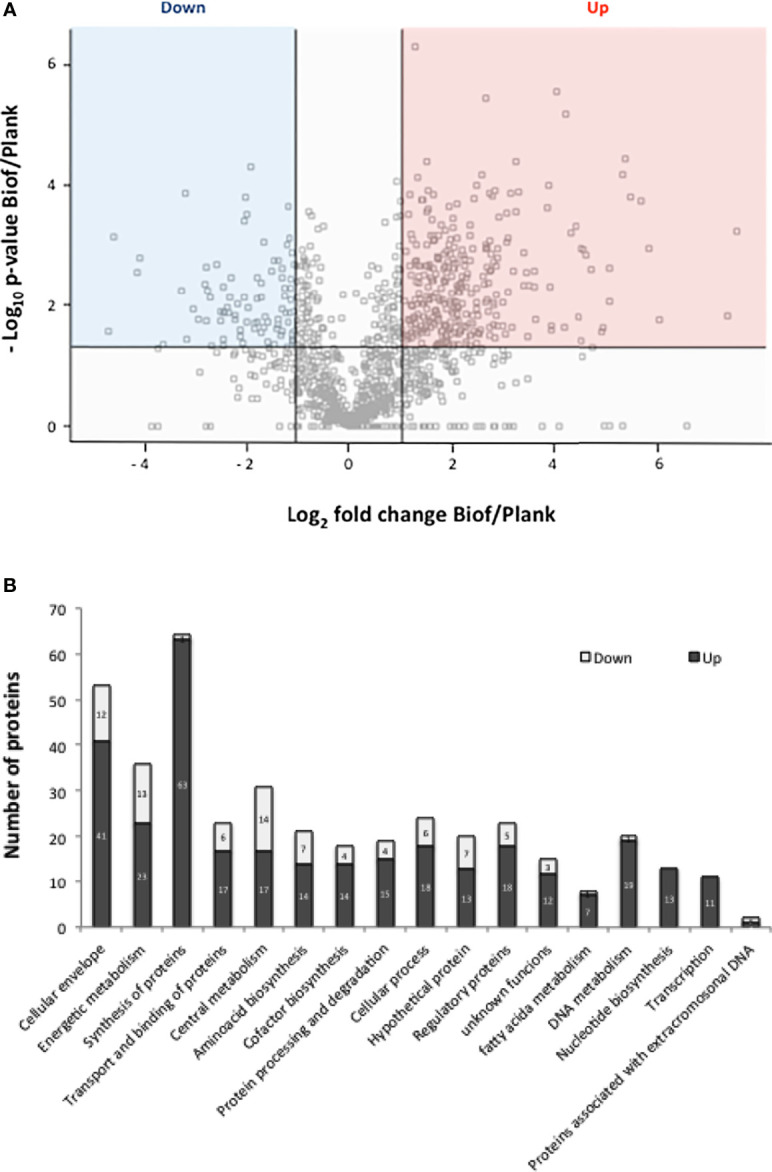
**(A)** Volcano plot of protein samples from the clinical isolate *B*. *pertussis* BpAR106 grown in biofilm (Biof) or planktonic (Plank) condition. -Log_10_ of the *p*-value associated with the magnitude of the fold-change of a given protein between the biofilm and planktonic growth conditions is plotted on the ordinate as a function of the log_2_ of that fold change on the abscissa. M = log_2_(fold-change). The overexpressed proteins are indicated in red (M ≥ 1), and the underexpressed proteins in blue (M ≤ −1) based on *p*<0.05. The proteins located on the 0 line correspond to the ON/OFF proteins since those proteins did not have an associated *p*-value. **(B)** Representation of the different clusters of orthologous groups (COGs) containing the proteins differentially expressed under biofilm culture condition of *B*. *pertussis* BpAR106. In the figure, the bar length represents the number of overexpressed (black) or underexpressed (gray) proteins within each COG among the total differentially expressed proteins.

To add evidence that the starting bacteria from which OMV were isolated actually came from biofilm lifestyle, we conducted a proteomic study to detect those differential proteins that were reported as characteristic of this lifestyle. Perseus software (version 1.5.6.0, Max Planck Institute, Germany) was used for the statistical analysis and visualization of the differences and common proteins obtained from biofilm or from planktonic cultures. The statistical significance of the relative quantitative changes between the growth conditions in biofilm *vs* planktonic cultures was determined by Student’s *t*-test at *p <*0.05. To assign a location to each protein in the respective routes, the Kyoto Encyclopedia of Genes and Genomes (KEGG) and STRING version 9.1 were used, in addition to protein-by-protein searches. In total, 1144 proteins were detected, of which 402 were found as differential between the two culture conditions analyzed. From these 402 proteins (the list of proteins is presented in **Table 1** of the **Supplementary Material**), 316 were overexpressed in biofilm, and 86 were overexpressed in planktonic culture (**Figure 3A**). Among the overexpressed proteins, BipA, fimbria, and other adhesins reported as marker of biofilm culture condition were detected (31, 46). In agreement to the findings reported by de Gouw et al. (32) and Dorji et al. (33), functional analysis using *B. pertussis* database showed that proteins involved in cell envelope, energy metabolism and protein synthesis were significantly overproduced in biofilm cells compared to planktonic cells (**Figure 3B**).

### Isolation and Immunogenic Characterization of OMV Derived From BpAR106 Grown in Biofilm

**Figure 4:**
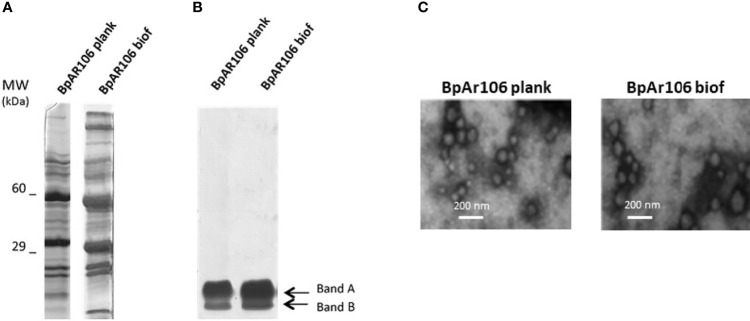
**(A)** SDS-PAGE (12.5%) of OMV derived from *B*. *pertussis* BpAR106 grown under planktonic or biofilm culture conditions. Molecular weights are indicated at the left. **(B)** Lipo-oligosaccharides content in OMVplank and OMVbiof suspensions normalized by total protein content. **(C)** Scanning electron micrographs of negative stained OMV obtained from *B*. *pertussis* BpAR106 grown under planktonic or biofilm culture conditions (scale bar: 200 nm).

We obtained OMV from BpAR106 in biofilm (OMVbiof) or planktonic (OMVplank) cultures using the protocol described by our group (20, 42). Similar unidimensional SDS-PAGE profiles of proteins (**Figure 4A**) and lipo-oligosaccharides (**Figure 4B**) were observed in extracts from OMVbiof and OMVplank. When observed under transmission electron microscopy, the sizes of both OMV were similar, and consistently with previously reported, had a size range between 50 and 250 nm (**Figure 4C**) (42). Then, we proceeded to the vaccine formulation of OMVbiof or OMVplank following the protocol described in the *Materials and Methods* section. To evaluate whether OMVbiof improved the protective capacity of OMVplank we used suboptimal protective OMV doses 0.75 μg of OMV (as total protein) per animal instead of the previously described protective dose of 3 μg (42, 44). This suboptimal protective dose was determined by the dose-response experiment showed in the **Supplementary Material** (**Figure S2**).

**Figure 5:**
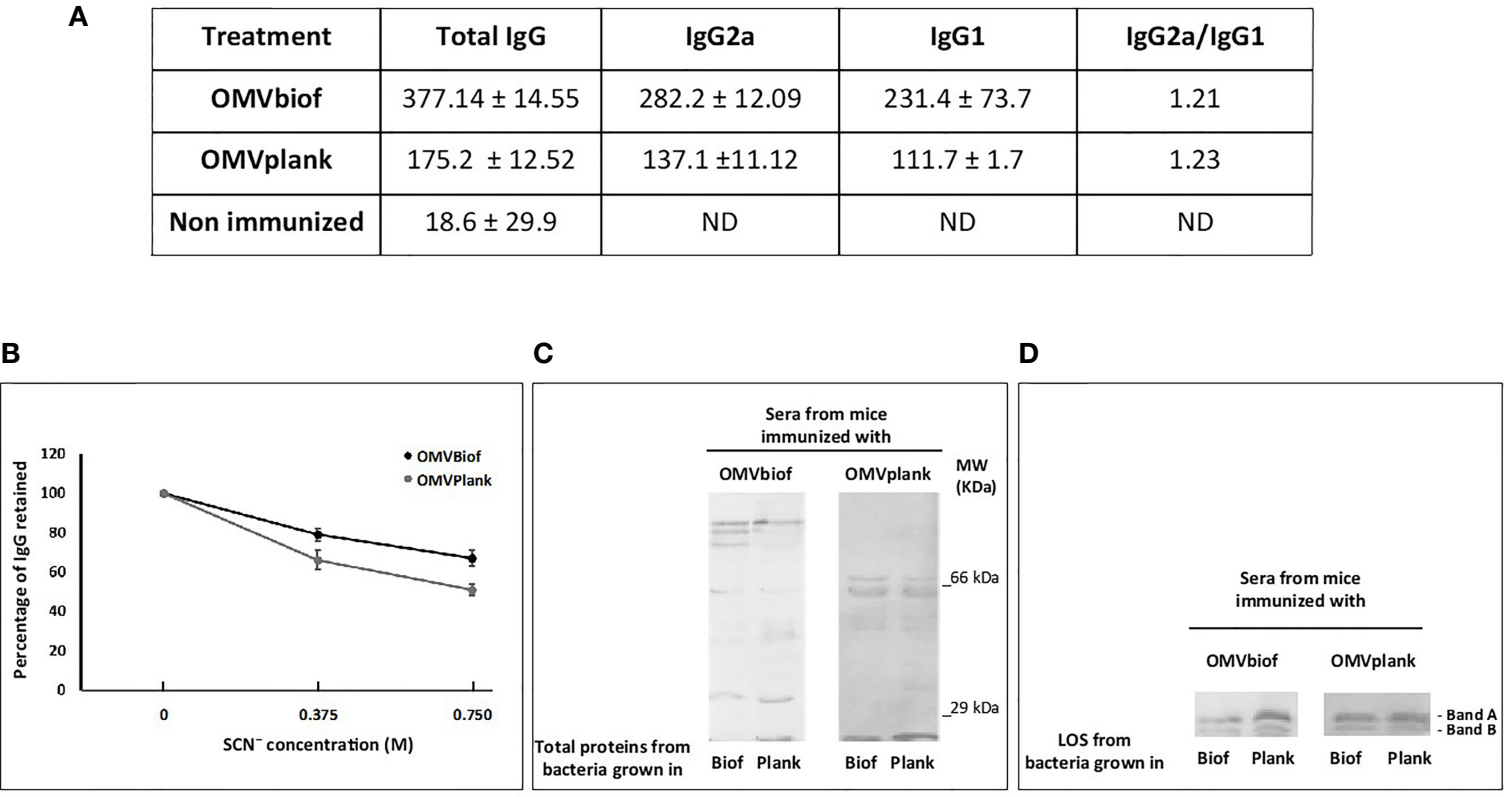
Anti-*B. pertussis* antibodies induced by 2-dose vaccination schedules. **(A)** Anti-*B. pertussis* IgG titers as well as the IgG isotypes were measured 14 days after the second vaccination dose. The titers are expressed as the geometric mean of the data from each group. **(B)** The avidity of IgG antibodies was also measured 14 days after the second dose and is represented as percentages of eluted *B*. *pertussis*-specific antibodies after treatment with increasing concentrations of ammonium thiocyanate (NH_4_SCN). The asterisk indicates statistically significant difference with p < 0.05. **(C)** Immunoblotting of total proteins of *B*. *pertussis* BpAR106 separated by 12.5% (w/v) SDS-PAGE and probed with the polyclonal antiserum obtained from immunized mice. The sera are designated according to the OMV-based vaccine used to immunize the mice. **(D)** Immunoblotting of BpAR106 LOS from both culture condition here tested separated by 12.5% (w/v) SDS-PAGE and probed with the polyclonal antiserum obtained from immunized mice. The sera are designated according to the OMV-based vaccine used to immunize the mice.

First, we evaluated the effect of a schedule consisting in 2 doses of vaccine on inducing specific IgG against *B. pertussis* (**Figure 5**). IgG2a and IgG1 levels were also evaluated after the second dose of each OMV. Higher values of IgG titers were detected in OMVbiof-immunized mice in comparison with OMVplank-immunized mice (*p*<0.05, **Figure 5A**). An interesting observation was the higher proportion of anti-Bp IgG with high avidity detected in the sera of OMVbiof-immunized mice (**Figure 5B**). In addition, we observed differential recognition patterns in blots from SDS-PAGE probed with the tested vaccine-induced sera (**Figure 5C**). When sera induced by OMVbiof are confronted with BpAR106 lysates obtained from biofilm or planktonic cultures, more complex recognition profiles were detected in comparison with the patterns detected with OMVplank-induced sera. As expected, the recognition of naïve animal sera was null (data not shown). Both OMVbiof and OMVplank-induced sera recognized the 2 characteristic electrophoretic bands (Band A and Band B) of the LOS extracted from both culture conditions (**Figure 5D**).

Moreover, both OMV triggered murine antibody responses with an IgG2a/IgG1 > 1.2 (**Figure 5A**) suggesting that both OMV skewed the immune response to a Th1 profile.

### Protective Capacity of OMVbiof Against Current Circulating Bacteria

**Figure 6:**
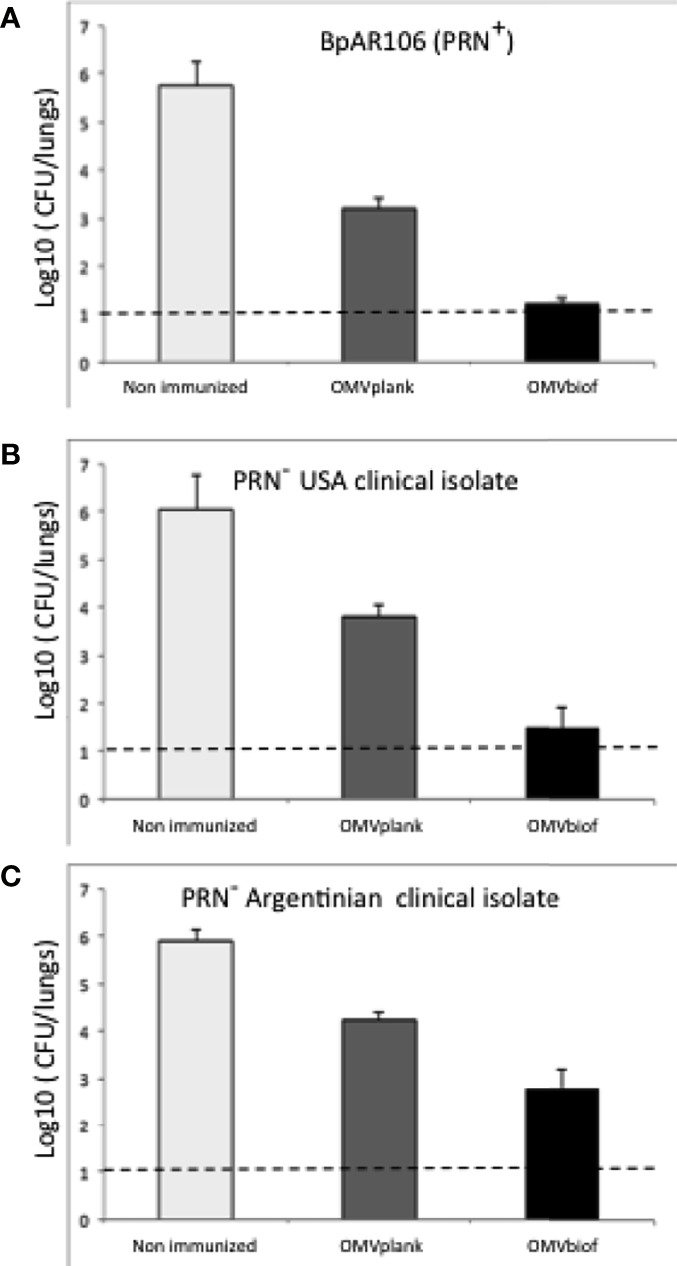
Protection against *B*. *pertussis* PRN(-) isolates induced with OMVbiof- or OMVplank-based vaccines in a mouse model. BALB/c mice were immunized (i.m) twice, 2 weeks apart. Mice were challenged with sublethal doses (5 x 10^7^ CFU/40 μl) of *B*. *pertussis* BpAR106 PRN(+) **(A)**, USA PRN(-) *B*. *pertussis* clinical isolate **(B)** or Argentinian PRN(-) B. pertussis clinical isolate **(C)**, 2 weeks after the second immunization with OMV-based vaccine. Non-immunized animals were included as negative control of protection. Three independent experiments were performed for each strain/isolate. Results from one representative experiment are shown. Results depicted are means of 5 mice per group sacrificed at 7 days post-challenge. The dashed line indicates the lower limit of detection. The number of bacteria recovered from mouse lungs is expressed as the average log_10_ CFU ± SEM (error bars) per lung. Data obtained were analyzed statistically by using one-way analysis of variance (ANOVA) followed by Bonferroni´s multiple comparison test (GraphPadPrism^®^). For panel **(A)** significant differences among the treatments with *p* < 0.001 were detected. For panels **(B, C)** significant differences among the treatments with *p* < 0.05 were detected.

To compare the protective capacity of OMVbiof and OMVplank vaccines, mice were immunized twice with each formulation and challenged with a sublethal dose of *B. pertussis* 2 weeks after the second immunization. For the first challenge we used BpAR106 bacteria expressing *prn* gene (*ptx*P3, *ptx*A1 and *prn*2, PRN+). As negative control of protection we used mice treated with PBS. Significant differences in lung CFU between immunized animals and negative controls were observed (*p*<0.001) (**Figure 6A**). While almost 2.5 log_10_ differences between OMVplank-vaccinated and non-immunized mice were detected, more than 4 log_10_ differences were observed between OMVbiof-vaccinated and non-immunized mice (**Figure 6A**).

When the challenge was performed with a PRN(-) clinical isolate obtained during an outbreak in USA (*ptx*P3, *ptx*A1 and *prn*2, PRN-), again the protective capacity of OMVbiof vaccine was significantly higher than that of OMVplank vaccine (**Figure 6B**). Mice immunized with the OMVbiof had more than 4 log_10_ reduction in lung CFU 7 days after challenge with *B. pertussis* PRN(-) compared with the non-immunized mice, whereas in the lungs of mice immunized with the OMVplank vaccine the reduction was only 2.5 log_10_ (p < 0.05, **Figure 6B**). Similar results were found against another PRN(-) clinical isolate obtained in Argentina: while OMVbiof reduced the number of lung CFU by 3 log_10_, OMVplank did it by 1.9 log_10_ (p < 0.05, **Figure 6C**).

## Discussion

In response to the need of a safer pertussis vaccine, a second generation of vaccine consisting in purified *B. pertussis* immunogens (aP vaccines) was developed. Thus, the first aP vaccines containing only pertussis toxin or pertussis toxin and filamentous hemagglutinin were licensed in the 80s (47). Current aP vaccines contain between two and five immunogens. These aP vaccines mainly induce Th2 response with poor induction of memory B-cells (48). On the other hand, T-cell responses were detected mainly in wP-primed children boosted by aP or natural infections but not in aP-primed children (49). The high concentration of immunogens in aP vaccines and their immune response not very robust seem to have contributed to the selection of *B. pertussis* strains more resistant to vaccination, favoring the emergence of genetically and phenotypically distinct *B. pertussis* strains (50). A novel P3 linage antigenically distinct from vaccine strains that produces higher levels of pertussis toxin is now common in most countries (51, 52). *B. pertussis* strains that are deficient in the production of the pertactin immunogen are prevalent in countries where only aP vaccines are used. In many of these countries the number of pertussis cases was increasing during the last decades (23, 53, 54).

To improve the epidemiological situation of pertussis, a third generation of acellular vaccines that stimulate a potent T-cell response, confer long lasting immunity and do not generate a selection pressure on the circulating bacterial population is necessary (10). In this context, our group has designed an OMV-based vaccine candidate that overcomes the weaknesses of current acellular vaccines (55). Preclinical tests in mice have shown that our vaccine candidate is safe and effective in avoiding the colonization of animals caused by different genotypes of *B. pertussis* (13, 44). We have also found that our candidate improves protection against pertactin-deficient isolates as compared to current commercial aP vaccines (13). The levels of protection detected for the OMV-based vaccine against these PRN(-) *B. pertussis* isolates are high; however, improvements to increase the reduction of pathogen colonization are still required to achieve protection levels similar as those detected for the PRN(+) *B. pertussis* isolates (13). Based on the knowledge that current circulating *B. pertussis* isolates form biofilms more readily than the strains used for vaccine production, and that all currently available pertussis vaccines are formulated with antigens derived from planktonic bacterial cells, an attractive improvement could be the obtaining of OMV from clinical isolate grown in biofilm culture. This hypothesis is also supported by the fact that proteins that are expressed only when bacteria grow in biofilms are immunogenic and possess immunoprotective capacity in the murine model (32). We prepared biofilms with a *B. pertussis* clinical isolate obtained in Argentina since these isolates were reported as good biofilm producers (30). This growth capability was evidenced here for BpAR106 clinical strain. Proteomic studies carried out with this strain comparing bacteria grown in biofilm with those obtained from planktonic culture showed that 316 proteins were overexpressed in the biofilm culture, among them BipA, fimbria, and other adhesins reported as biofilm markers (31, 46). In addition, we detected OMV within the *B. pertussis* biofilm, with a size range similar to OMV obtained from *B. pertussis* planktonic cultures. This finding raises questions about the OMV formation mechanism and its role within the biofilm, as well as about biofilm formation *in vivo*. Though these topics are interesting, they were not addressed in this work since our objective was focused on obtaining OMVbiof to improve our vaccine candidate protective capacity.

The vaccine formulated with OMVbiof obtained from *B. pertussis* BpAR106 was more immunogenic than the OMVplank vaccine candidate not only in terms of specific antibody titers but also in their quality (higher avidity). Furthermore, the formulation based on OMVbiof induced a mixed Th1 and Th2 profile according to the IgG2a/IgG1 ratio detected and was more protective in mice assays challenged with the PRN(+) *B. pertussis* clinical isolate. This higher immunogenicity is in agreement with earlier findings by Dorji et al. (34) who demonstrated in mice model that proteins typical of bacteria grown in biofilm led to a more Th1-type response profile when added to commercial aP vaccines and additionally, retained lower bacterial loads than mice vaccinated with aP alone (34). Moreover, de Gouw et al. also shown that BipA, a biofilm characteristic protein, induces a significant reduction in colonization of mice lungs (56). These authors also detected that membrane proteins derived from biofilm bacteria induced protection against respiratory *B. pertussis* infection. Lipoproteins and Fim chaperones, in addition to the LOS adjuvant action, are also expected to play a role in the induction of protection. Regarding the LOS molecule, here we detected LOS specific antibodies induced by OMV-based vaccines as was previously reported by Raeven et al. (57). Previous results showed that antibodies against LOS, detected in the sera from individuals convalescing from natural infection, have bactericidal activity (58). These results showed the importance of LOS presence in the new generation of pertussis vaccines in lower quantities than in the wP vaccine, as occurred in the case of OMV-based formulation. Other non-excluding alternative might be the use of formulation with LOS containing modified lipid A (42) since antibodies to the terminal trisaccharide appear to be sufficient to elicit bactericidal activity (58).

The boundary condition of suboptimal OMV amounts to induce protection allowed us to detect the impact of OMVbiof on improvement in vaccine design. With this approach, we also showed that OMVbiof are superior in terms of protective capacity against PRN(-) isolates, which are currently prevalent in countries that use only aP vaccines in their calendars. Thus, protection level detected of OMVbiof against both PRN(-) clinical isolates (obtained in USA or in Argentina) not only were higher than those observed for OMVplank but also were within the accepted protection standard for a pertussis vaccine.

The representativeness of a given *B. pertussis* isolate from which a new vaccine is generated might be narrow and, above all, limited in time. The attempt to overcome this is that the growth in biofilm by itself has characteristics that seem to go beyond the specificity of each isolate and that may cushion the differences. The broad protective capacity shown in **Figure 6** would be in line with this idea. An alternative might be the use of a mixture of OMV from different strains.

Undoubtedly, all these data position OMVbiof as an interesting approach to overcome the weaknesses of current commercial vaccines formulated with immunogens obtained from planktonic cultures, which do not necessarily reflect the composition and physiology of the pathogen during the *in vivo* infection.

## Data Availability Statement

The raw data supporting the conclusions of this article will be made available by the authors, without undue reservation.

## Ethics Statement

The animal study was reviewed and approved by Ethical Committee for Animal Experiments of the Faculty of Exact Sciences of UNLP (Argentina, approval number 004-06-15 and 003-06-15).

## Author Contributions

DH planned the study, made the laboratory analysis, interpreted data, and drafted manuscript. DB, EG, EZ, and AL interpreted data, and revised figures and the manuscript. FC, PA, NA, CC, and ER performed certain experiments and laboratory analyses. All authors contributed to the article and approved the submitted version.

## Funding

This work was supported by ANCPyT (PICT 2014-3617, PICT 2017- 2365), CONICET grants to DH. DH, EG, EZ, DB, and AL are members of the Scientific Career of CONICET. FC, PA, and ER are fellows from CONICET. NA is a fellow from ANPCyT and CC is a full-time Professor at Universidad Nacional de La Plata.

## Conflict of Interest

The authors declare that the research was conducted in the absence of any commercial or financial relationships that could be construed as a potential conflict of interest.

## Publisher’s Note

All claims expressed in this article are solely those of the authors and do not necessarily represent those of their affiliated organizations, or those of the publisher, the editors and the reviewers. Any product that may be evaluated in this article, or claim that may be made by its manufacturer, is not guaranteed or endorsed by the publisher.

## References

[1] Tan T Dalby T Forsyth K Halperin SA Heininger U Hozbor D. Pertussis Across the Globe: Recent Epidemiologic Trends From 2000 to 2013. Pediatr Infect Dis J (2015) 34:e222–32. doi: 10.1097/INF.0000000000000795 26376316

[2] Syed MA Bana NF. Pertussis. A Reemerging and an Underreported Infectious Disease. Saudi Med J (2014) 35:1181–7. PMC4362115 25316461

[3] Jakinovich A Sood SK. Pertussis: Still a Cause of Death, Seven Decades Into Vaccination. Curr Opin Pediatr (2014) 26:597–604. doi: 10.1097/MOP.0000000000000139 25136948

[4] Domenech de Cellès M Magpantay FMG King AA Rohani P. The Pertussis Enigma: Reconciling Epidemiology, Immunology and Evolution. Proc Biol Sci (2016) 283:2015–309. doi: 10.1098/rspb.2015.2309 PMC4721090 26763701

[5] Damron FH Barbier M Dubey P Edwards KM Gu X-X Klein NP. Overcoming Waning Immunity in Pertussis Vaccines: Workshop of the National Institute of Allergy and Infectious Diseases. J Immunol (Baltimore Md: 1950) (2020) 205:877–82. doi: 10.4049/jimmunol.2000676 PMC7454230 32769142

[6] de Miranda Lopes KA Baptista PN de Medeiros Nascimento R Pimentel A de Alencar Ximenes RA. Clinical Repercussions in Pertussis Infants Post-Tdpa Vaccination of Pregnant Woman: An Immunization Success? Vaccine (2021) 39:2555–60. doi: 10.1016/j.vaccine.2021.03.069 33814232

[7] Riccò M Vezzosi L Gualerzi G Bragazzi NL Balzarini F. Pertussis Immunization in Healthcare Workers Working in Pediatric Settings: Knowledge, Attitudes and Practices (KAP) of Occupational Physicians. Preliminary Results From a Web-Based Survey (2017). J Prev Med Hygiene (2020) 61:E66–75. doi: 10.15167/2421-4248/jpmh2020.61.1.1155 PMC7225653 32490271

[8] Saadatian-Elahi M Plotkin S Mills KHG Halperin SA McIntyre PB Picot V. Pertussis: Biology, Epidemiology and Prevention. Vaccine (Elsevier Ltd) (2016) 5819–26. doi: 10.1016/j.vaccine.2016.10.029 27780629

[9] Mills KHG Ross PJ Allen AC Wilk MM. Do We Need a New Vaccine to Control the Re-Emergence of Pertussis? Trends Microbiol (2016) 22:49–52. doi: 10.1016/j.tim.2013.11.007 24485284

[10] Hozbor D. New Pertussis Vaccines: A Need and a Challenge. In: Advances in Experimental Medicine and Biology. Springer (2019). p. 115–26. doi: 10.1007/5584_2019_407 31432399

[11] Locht C. Pertussis: Where did We Go Wrong and What can We do About it? J Infect (2016) 72 Suppl:S34–40. doi: 10.1016/j.jinf.2016.04.020 27161992

[12] Brummelman J Wilk MM Han WGH van Els CACM Mills KHG. Roads to the Development of Improved Pertussis Vaccines Paved by Immunology. Pathog Dis (2015) 73:ftv067. doi: 10.1093/femspd/ftv067 26347400 PMC4626578

[13] Zurita ME Wilk MM Carriquiriborde F Bartel E Moreno G Misiak A. A Pertussis Outer Membrane Vesicle-Based Vaccine Induces Lung-Resident Memory CD4 T Cells and Protection Against Bordetella Pertussis, Including Pertactin Deficient Strains. Front Cell Infection Microbiol (2019) 9:125. doi: 10.3389/fcimb.2019.00125 PMC6498398 31106160

[14] Wilk MM Misiak A Mcmanus RM Allen AC Lynch MA Mills KHG. Lung CD4 Tissue-Resident Memory T Cells Mediate Adaptive Immunity Induced by Previous Infection of Mice With Bordetella Pertussis. J Immunol (2017) 199:233–43. doi: 10.4049/jimmunol.1602051 28533445

[15] Diavatopoulos DA Mills KHG Kester KE Kampmann B Silerova M Heininger U. PERISCOPE: Road Towards Effective Control of Pertussis. Lancet Infect Dis (2019) 19:e179–86. doi: 10.1016/S1473-3099(18)30646-7 30503084

[16] Bottero D Gaillard ME Basile LA Fritz M Hozbor DF. Genotypic and Phenotypic Characterization of Bordetella Pertussis Strains Used in Different Vaccine Formulations in Latin America. J Appl Microbiol (2012) 112:1266–76. doi: 10.1111/j.1365-2672.2012.05299.x 22471652

[17] Parkhill J Sebaihia M Preston A Murphy LD Thomson N Harris DE. Comparative Analysis of the Genome Sequences of Bordetella Pertussis, Bordetella Parapertussis and Bordetella Bronchiseptica. Nat Genet (2003) 35:32–40. doi: 10.1038/ng1227 12910271

[18] Caro V Bouchez V Guiso N. Is the Sequenced Bordetella Pertussis Strain Tohama I Representative of the Species? J Clin Microbiol (2008) 46:2125–8. doi: 10.1128/JCM.02484-07 PMC2446832 18385436

[19] Hozbor DF. Outer Membrane Vesicles: An Attractive Candidate for Pertussis Vaccines. Expert Rev Vaccines (2017) 16:193–6. doi: 10.1080/14760584.2017.1276832 28010142

[20] Roberts R Moreno G Bottero D Gaillard ME Fingermann M Graieb A. Outer Membrane Vesicles as Acellular Vaccine Against Pertussis. Vaccine (2008) 26:4639–46. doi: 10.1016/j.vaccine.2008.07.004 18640169

[21] Elizagaray ML Gomes MTR Guimaraes ES Rumbo M Hozbor DF Oliveira SC. Canonical and Non-Canonical Inflammasome Activation by Outer Membrane Vesicles Derived From Bordetella Pertussis. Front Immunol (2020) 11:1879. doi: 10.3389/fimmu.2020.01879 32973778 PMC7468456

[22] Gaillard ME Bottero D Errea A Ormazábal M Zurita ME Moreno G. Acellular Pertussis Vaccine Based on Outer Membrane Vesicles Capable of Conferring Both Long-Lasting Immunity and Protection Against Different Strain Genotypes. Vaccine (2014) 32:931–7. doi: 10.1016/j.vaccine.2013.12.048 24397896

[23] Martin SW Pawloski L Williams M Weening K Debolt C Qin X. Pertactin-Negative Bordetella Pertussis Strains: Evidence for a Possible Selective Advantage. Clin Infect Dis (2015) 60:223–7. doi: 10.1093/cid/ciu788 25301209

[24] S B AT S. Analysis of Bordetella Pertussis Pertactin and Pertussis Toxin Types From Queensland, Australia, 1999-2003. BMC Infect Dis (2006) 6:53–61. doi: 10.1186/1471-2334-6-53 16542440 PMC1459169

[25] Weigand MR Williams MM Peng Y Kania D Pawloski LC Tondella ML. Genomic Survey of Bordetella Pertussis Diversity, United States, 2000–2013. Emerging Infect Dis (2019) 25:780–3. doi: 10.3201/eid2504.180812 PMC6433035 30882317

[26] Zomer A Otsuka N Hiramatsu Y Kamachi K Nishimura N Ozaki T. Bordetella Pertussis Population Dynamics and Phylogeny in Japan After Adoption of Acellular Pertussis Vaccines. Microbial Genomics (2018) 4:180–93. doi: 10.1099/mgen.0.000180 PMC5994715 29771235

[27] Carriquiriborde F Regidor V Aispuro PM Magali G Bartel E Bottero D. Rare Detection of Bordetella Pertussis Pertactin-Deficient Strains in Argentina. Emerging Infect Dis (2019) 25:2048–54. doi: 10.3201/eid2511.190329 PMC6810201 31625838

[28] Safarchi A Octavia S Luu LDW Tay CY Sintchenko V Wood N. Pertactin Negative Bordetella Pertussis Demonstrates Higher Fitness Under Vaccine Selection Pressure in a Mixed Infection Model. Vaccine (2015) 33:6277–81. doi: 10.1016/j.vaccine.2015.09.064 26432908

[29] Hegerle N Dore G Guiso N. Pertactin Deficient Bordetella Pertussis Present a Better Fitness in Mice Immunized With an Acellular Pertussis Vaccine. Vaccine (2014) 32:6597–600. doi: 10.1016/j.vaccine.2014.09.068 25312274

[30] Arnal L Grunert T Cattelan N de Gouw D Villalba MI Serra DO. Bordetella Pertussis Isolates From Argentinean Whooping Cough Patients Display Enhanced Biofilm Formation Capacity Compared to Tohama I Reference Strain. Front Microbiol (2015) 6:1352. doi: 10.3389/fmicb.2015.01352 26696973 PMC4672677

[31] Cattelan N Jennings-Gee J Dubey P Yantorno OM Deora R. Hyperbiofilm Formation by Bordetella Pertussis Strains Correlates With Enhanced Virulence Traits. Infection Immun (2017) 85:373–15. doi: 10.1128/IAI.00373-17 PMC5695122 28893915

[32] de Gouw D Serra DO de Jonge MI Hermans PW Wessels HJ Zomer A. The Vaccine Potential of Bordetella Pertussis Biofilm-Derived Membrane Proteins. Emerging Microbes infections (2014) 3:e58. doi: 10.1038/emi.2014.58 PMC4150286 26038752

[33] Dorji D Graham RM Richmond P Keil A Mukkur TK. Biofilm Forming Potential and Antimicrobial Susceptibility of Newly Emerged Western Australian Bordetella Pertussis Clinical Isolates. Biofouling (2016) 32:1141–52. doi: 10.1080/08927014.2016.1232715 27669900

[34] Dorji D Graham RM Singh AK Ramsay JP Price P Lee S. Immunogenicity and Protective Potential of Bordetella Pertussis Biofilm and Its Associated Antigens in a Murine Model. Cell Immunol (2019) 337:42–7. doi: 10.1016/j.cellimm.2019.01.006 30770093

[35] Bottero D Gaillard ME Fingermann M Weltman G Fernández J Sisti F. Pulsed-Field Gel Electrophoresis, Pertactin, Pertussis Toxin S1 Subunit Polymorphisms, and Surfaceome Analysis of Vaccine and Clinical Bordetella Pertussis Strains. Clin Vaccine Immunol (2007) 14:1490–8. doi: 10.1128/CVI.00177-07 PMC2168178 17699837

[36] Pawloski LC Queenan AM Cassiday PK Lynch AS Harrison MJ Shang W. Prevalence and Molecular Characterization of Pertactin-Deficient Bordetella Pertussis in the United States. Clin Vaccine Immunol (2014) 21:119–25. doi: 10.1128/CVI.00717-13 PMC3910938 24256623

[37] Von Koenig CHW Tacken A Finger H. Use of Supplemented Stainer-Scholte Broth for the Isolation of Bordetella Pertussis From Clinical Material. J Clin Microbiol (1988) 26:2558–60. doi: 10.1128/jcm.26.12.2558-2560.1988 PMC266945 2906641

[38] Stainer DW Scholte MJ. A Simple Chemically Defined Medium for the Production of Phase I Bordetella Pertussis. J Gen Microbiol (1970) 63:211–20. doi: 10.1099/00221287-63-2-211 4324651

[39] O’Toole GA. Microtiter Dish Biofilm Formation Assay. J Visualized Experiments (2010) 47:2437–8. doi: 10.3791/2437 PMC3182663 21307833

[40] Hozbor D Rodriguez ME Fernández J Lagares A Guiso N Yantorno O. Release of Outer Membrane Vesicles From Bordetella Pertussis. Curr Microbiol (1999) 38:273–8. doi: 10.1007/PL00006801 10355115

[41] Kielkopf CL Bauer W Urbatsch IL. Bradford Assay for Determining Protein Concentration. Cold Spring Harbor Protoc (2020) 2020:136–8. doi: 10.1101/pdb.prot102269 32238597

[42] Asensio CJA Gaillard ME Moreno G Bottero D Zurita E Rumbo M. Outer Membrane Vesicles Obtained From Bordetella Pertussis Tohama Expressing the Lipid a Deacylase PagL as a Novel Acellular Vaccine Candidate. Vaccine (2011) 29:1649–56. doi: 10.1016/j.vaccine.2010.12.068 21211579

[43] Nilsson JF Castellani LG Draghi WO Pérez-Giménez J Torres Tejerizo GA Pistorio M. Proteomic Analysis of Rhizobium Favelukesii LPU83 in Response to Acid Stress. J Proteome Res (2019) 18:3615–29. doi: 10.1021/acs.jproteome.9b00275 31432679

[44] Bottero D Gaillard ME Zurita E Moreno G Martinez DS Bartel E. Characterization of the Immune Response Induced by Pertussis OMVs-Based Vaccine. Vaccine (2016) 34:3303–9. doi: 10.1016/j.vaccine.2016.04.079 27151884

[45] Bart MJ Harris SR Advani A Arakawa Y Bottero D Bouchez V. Global Population Structure and Evolution of Bordetella Pertussis and Their Relationship With Vaccination. mBio (2014) 5:e01074-14. doi: 10.1128/mBio.01074-14 PMC3994516 24757216

[46] Serra DO Lücking G Weiland F Schulz S Görg A Yantorno OM. Proteome Approaches Combined With Fourier Transform Infrared Spectroscopy Revealed a Distinctive Biofilm Physiology in Bordetella Pertussis. Proteomics (2008) 8:4995–5010. doi: 10.1002/pmic.200800218 18972542

[47] Sato Y Kimura M Fukumi H. Development of a Pertussis Component Vaccine In Japan. Lancet (1984) 323:122–6. doi: 10.1016/S0140-6736(84)90061-8 6140441

[48] Edwards KM Berbers GAM. Immune Responses to Pertussis Vaccines and Disease. J Infect Dis (2014) 209:s10-5. doi: 10.1093/infdis/jit560 24158958

[49] Schure RM Hendrikx LH de Rond LGH Öztürk K Sanders EAM Berbers GAM. Differential T- and B-Cell Responses to Pertussis in Acellular Vaccine-Primed *Versus* Whole-Cell Vaccine-Primed Children 2 Years After Preschool Acellular Booster Vaccination. Clin Vaccine Immunol (2013) 20:1388–95. doi: 10.1128/CVI.00270-13 PMC3889587 23825195

[50] Octavia S Maharjan RP Sintchenko V Stevenson G Reeves PR Gilbert GL. Insight Into Evolution of Bordetella Pertussis From Comparative Genomic Analysis: Evidence of Vaccine-Driven Selection. Mol Biol Evol (2011) 28:707–15. doi: 10.1093/molbev/msq245 20833694

[51] Loconsole D de Robertis AL Morea A Metallo A Lopalco PL Chironna M. Resurgence of Pertussis and Emergence of the Ptx P3 Toxin Promoter Allele in South Italy. Pediatr Infect Dis J (2018) 37:E126–31. doi: 10.1097/INF.0000000000001804 28945679

[52] Armstrong ME Loscher CE Lynch MA Mills KHG. IL-1β-Dependent Neurological Effects of the Whole Cell Pertussis Vaccine: A Role for IL-1-Associated Signalling Components in Vaccine Reactogenicity. J Neuroimmunology (2003) 136:25–33. doi: 10.1016/S0165-5728(02)00468-X 12620640

[53] Barkoff AM He Q. Molecular Epidemiology of Bordetella Pertussis. In: Advances in Experimental Medicine and Biology. Springer (2019). p. 19–33. doi: 10.1007/5584_2019_402 31342459

[54] Fingermann M Fernández J Sisti F Rodríguez ME Gatti B Bottero D. Differences of Circulating Bordetella Pertussis Population in Argentina From the Strain Used in Vaccine Production. Vaccine (2006) 24:3513–21. doi: 10.1016/j.vaccine.2006.02.026 16545509

[55] Rumbo M Hozbor D. Development of Improved Pertussis Vaccine. Hum Vaccines Immunotherapeutics (2014) 10:2450–3. doi: 10.4161/hv.29253 PMC4896757 25424954

[56] de Gouw D de Jonge MI Hermans PWM Wessels HJCT Zomer A Berends A. Proteomics-Identified Bvg-Activated Autotransporters Protect Against Bordetella Pertussis in a Mouse Model. PloS One (2014) 9:e105011-19. doi: 10.1371/journal.pone.0105011 PMC4136822 25133400

[57] RH R van der LM T W U JP B TH K B. Immunoproteomic Profiling of Bordetella Pertussis Outer Membrane Vesicle Vaccine Reveals Broad and Balanced Humoral Immunogenicity. J Proteome Res (2015) 14:2929–42. doi: 10.1021/ACS.JPROTEOME.5B00258 25988566

[58] Weiss AA Mobberley PS Fernandez RC Mink CM. Characterization of Human Bactericidal Antibodies to Bordetella Pertussis. Infection Immun (1999) 67:1424. doi: 10.1128/IAI.67.3.1424-1431.1999 PMC96476 10024590

