# Supplementary material for: Pertussis Vaccine Candidate Based on Outer Membrane Vesicles Derived From Biofilm Culture
Source: Front Immunol. 2021 Sep 15;12:730434. doi: 10.3389/fimmu.2021.730434 (PMC8479151; doi:10.3389/fimmu.2021.730434)
Supplement: Supplementary file 1 [file DataSheet_1.pdf]

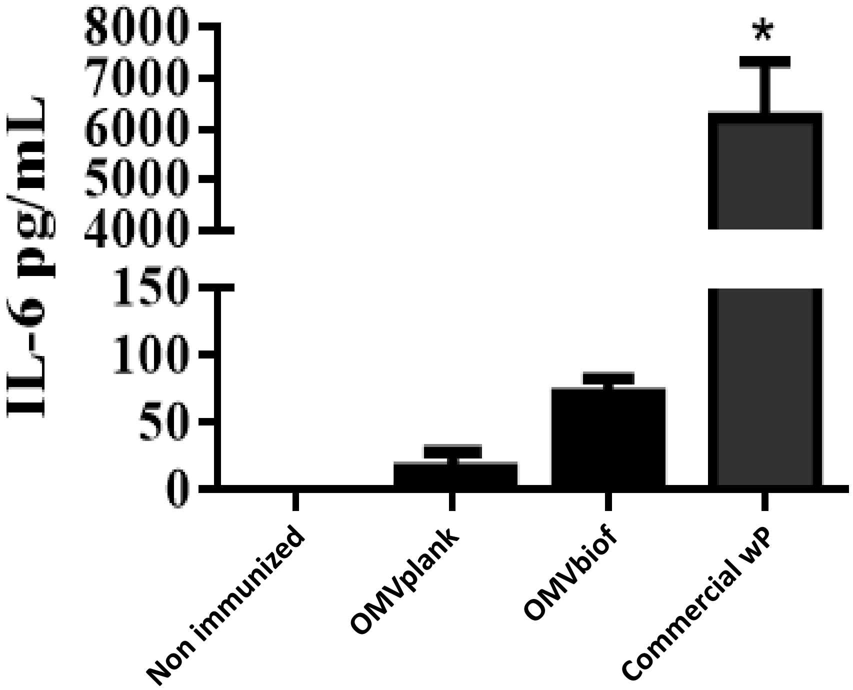

Fig S1 IL-6 production in sera of treated mice. Average results of 3 assays comparing the responses to the commercial vaccines are shown. The bars represent the standard errors of the geometric means. *p<0.001

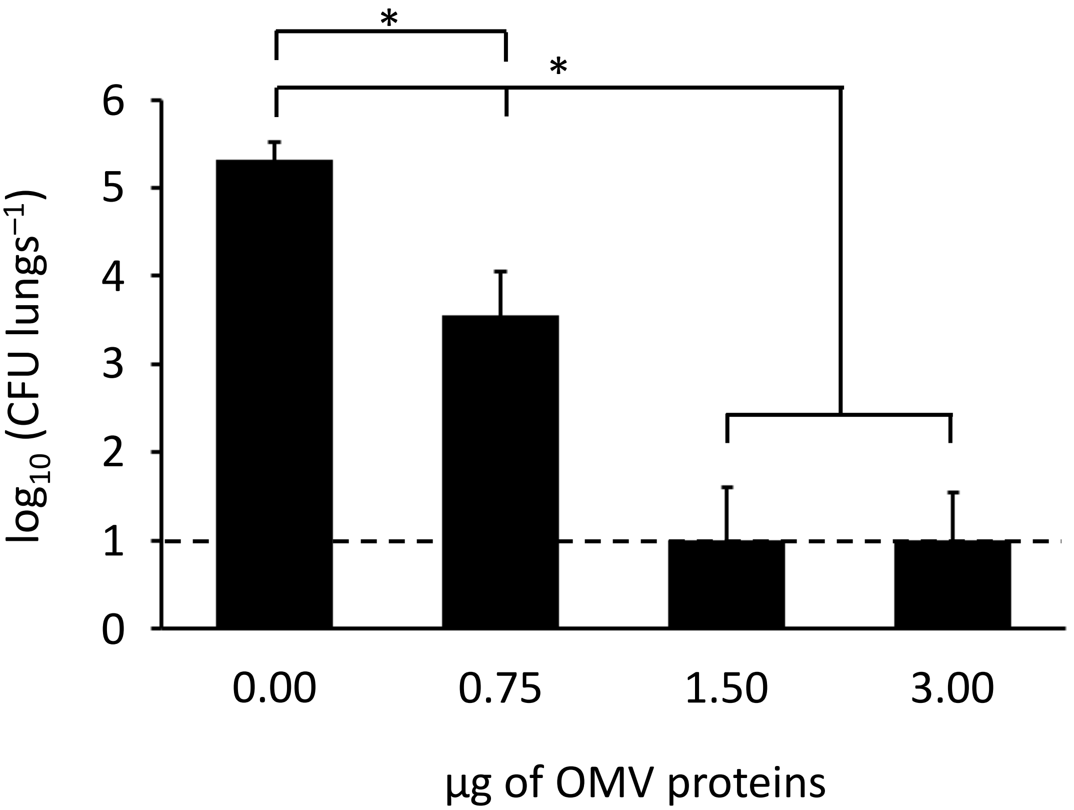

Fig S2 Protection against *B. pertussis* infection induced by vaccines containing different quantities of OMV (measured as total OMV proteins) in a mouse model. BALB/c mice were immunized (i.m) twice, 2 weeks apart. Mice were challenged with sublethal doses (5 x 10^7 CFU/40 µl) of *B. pertussis* 2 weeks after the second immunization with OMV-based vaccine. Non-immunized animals (0.00 µg of OMV proteins) were included as negative control of protection. Two independent experiments were performed. Results from one representative experiment are shown. Results depicted are means of 5 mice per group sacrificed at 7 days post-challenge. The dashed line indicates the lower limit of detection. The number of bacteria recovered from mouse lungs is expressed as the average $\log_{10}$ CFU $\pm$ SEM (error bars) per lung. Data obtained were analyzed statistically by using one-way analysis of variance (ANOVA) followed by Bonferroni's multiple comparison test (GraphPadPrism®). Statistical significant differences with $p<0.05$ are indicated by asterisks.
